# MGDB: a comprehensive database of genes involved in melanoma

**DOI:** 10.1093/database/bav097

**Published:** 2015-09-30

**Authors:** Di Zhang, Rongrong Zhu, Hanqian Zhang, Chun-Hou Zheng, Junfeng Xia

**Affiliations:** ^1^Institute of Health Sciences, School of Computer Science and Technology,; ^2^College of Electrical Engineering and Automation and; ^3^Center of Information Support and Assurance Technology, Anhui University, Hefei, Anhui 230601, China

## Abstract

The Melanoma Gene Database (MGDB) is a manually curated catalog of molecular genetic data relating to genes involved in melanoma. The main purpose of this database is to establish a network of melanoma related genes and to facilitate the mechanistic study of melanoma tumorigenesis. The entries describing the relationships between melanoma and genes in the current release were manually extracted from PubMed abstracts, which contains cumulative to date 527 human melanoma genes (422 protein-coding and 105 non-coding genes). Each melanoma gene was annotated in seven different aspects (General Information, Expression, Methylation, Mutation, Interaction, Pathway and Drug). In addition, manually curated literature references have also been provided to support the inclusion of the gene in MGDB and establish its association with melanoma. MGDB has a user-friendly web interface with multiple browse and search functions. We hoped MGDB will enrich our knowledge about melanoma genetics and serve as a useful complement to the existing public resources.

**Database URL:** http://bioinfo.ahu.edu.cn:8080/Melanoma/index.jsp

## Introduction

Melanoma is a malignant tumor that develops from specialized pigment cells called melanocytes and considered as one of the most aggressive human cancers. The incidence of melanoma is increasing at a rate faster than any other solid malignancy, with an estimated 232 100 new cases and 55 000 deaths reported worldwide in 2012 (1). Due to its aggressive behavior and limited treatment options, the overall survival for patients with metastatic melanoma is poor. Early detection is critical to improve the overall prognosis of melanoma and an important way to reduce deaths from melanoma.

Similar to many other cancers, melanoma is initiated by activation of oncogenes or inactivation of tumor suppressor genes. Recent advances in molecular profiling and genome sequencing have revealed that the melanoma is a genetic heterogeneous group of neoplasms whose progression is driven by distinct patterns of oncogenic mutation (2–4). Mutations in BRAF (at position V600) and NRAS (G12/13, Q61) are responsible for 50 and 20% of melanomas, respectively (5). Other drive genes involved include KIT (W557, V559, L576, K642, D816), GNAQ (Q209), and GNA11 (Q209) (5). These driver mutations confer growth and survival advantages on melanoma cells. The ability to identify driver genes, even when pharmacologic agents are not available, has stimulated interest in diagnostic and therapeutic strategies targeting molecular mechanisms (6). For example, the kinase inhibitors vemurafenib and imatinib have become standard treatments for patient whose tumor harbor driver mutations in BRAF or KIT, respectively. However, treatment options remain limited for the patients that lack mutations in the five most commonly mutated melanoma genes (BRAF, NRAS, KIT, GNAQ and GNA11) and the prognosis of such patients is particularly pool with a median overall survival of less than 1 year (7). Continued investigation for novel driver mutations and melanoma genes is critical to improve therapeutic outcomes for melanoma patients. For example, we previously determined that approximately 8% of melanomas negative for known drivers harbor other activating mutations in BRAF exon 15 (D594E/G/H/N/V, L597R/S/Q/V and K601E/I/N) rather than the well-known V600 mutation, and we showed that a patient with BRAF L597S mutation responded signicantly to treatment with the MEK inhibitor (8). More recently, we systematically examined mutations from published melanoma next generation sequencing (NGS) data and identied several potentially clinically relevant mutations (9).

In the last few years, a number of gene databases have emerged with a focus on a specialized cancer type as exemplified by Breast Cancer Gene Database (10), Oral Cancer Database (11), Prostate cancer (12), Lung Cancer Database (13), Cervical Cancer Gene Database (14) and Renal Cancer Gene Database (15), etc. However, there is no database focusing on genes involved in melanoma causation yet available. Therefore, we have collected melanoma related genes to construct an integrated database termed Melanoma Gene Database (MGDB) that catalogs the genes known to be involved in melanoma carcinogenesis as evidenced from the biomedical literature. To gather and uniformly present the available information on melanoma genes, we have created a user-friendly interface in the form of MGDB. The database integrates heterogeneous data including basic gene information, sequencing information from NCBI, manually curated literature references to support inclusion of a gene in the database, and detailed annotations for each melanoma genes, such as gene expressions from hundreds of tumor samples from the Cancer Genome Atlas (TCGA) project webpage (http://cancergenome.nih.gov), methylation data from GEO database (16), somatic mutations from COSMIC (17), interacting partners from PINA (18), pathway information from KEGG (19), and drug information from DGIdb (20). In addition, the database provides search facility for querying the database and an online BLAST interface for sequence search. It serves multiple purposes for the user, including: (i) obtaining literature-based gene lists for melanoma; (ii) browsing detailed information, including gene expression levels, methylation levels, gene mutation sites, protein–protein interactions, involved pathways and drug information; (iii) a resource for therapy melanoma; (iv) a resource for high-throughput genetic screening to find melanoma-related gene variants. Overall, MGDB serves as the most comprehensive data resource that will enable the exploration of relevant information for human melanoma related genes, and would be helpful for the research community.

## Data collection and content

To obtain a complete list of publications for melanoma genes, we made a comprehensive search for melanoma-related genetic studies in NCBI PubMed. Initially, we performed an extensive literature query of PubMed on 8 April 2015 using the following keywords combinations: ‘melanoma’ [Title/Abstract] OR ‘melanocyte’ [Title/Abstract] OR ‘melanin’ [Title/Abstract] OR ‘skin cancer’ [Title/Abstract]) AND (‘gene’ [Title/Abstract] AND (‘oncogene’ [Title/Abstract] OR ‘tumor’ [Title/Abstract] OR ‘suppressor’ [Title/Abstract] OR ‘neoplasm’ [Title/Abstract] OR ‘mutation’ [Title/Abstract] OR ‘expression’ [Title/Abstract] OR ‘microarray’ [Title/Abstract] OR ‘association’ [Title/Abstract] OR ‘linkage’ [Title/Abstract] OR ‘sequencing’ [Title/Abstract] OR ‘lncRNA’ [Title/Abstract]).

Overall, we obtained 9788 melanoma-related publications. The abstracts of these articles were manually screened, and those that studied other diseases instead of melanoma or those with negative results were excluded. In all, a total of 997 PubMed abstracts were collected and downloaded for further manual review and curation.

We then extracted descriptions of melanoma genes from abstracts and mapped the gene names to Entrez gene IDs, which was used to serve as the initial information to crosslink the same genes from different public databases. After carefully checking manually, we finally obtained 527 human melanoma genes (422 protein-coding, 95 microRNAs, and 10 lncRNAs) from 682 PubMed abstracts (Supplementary Tables S1 and S2).

**Figure 1.**
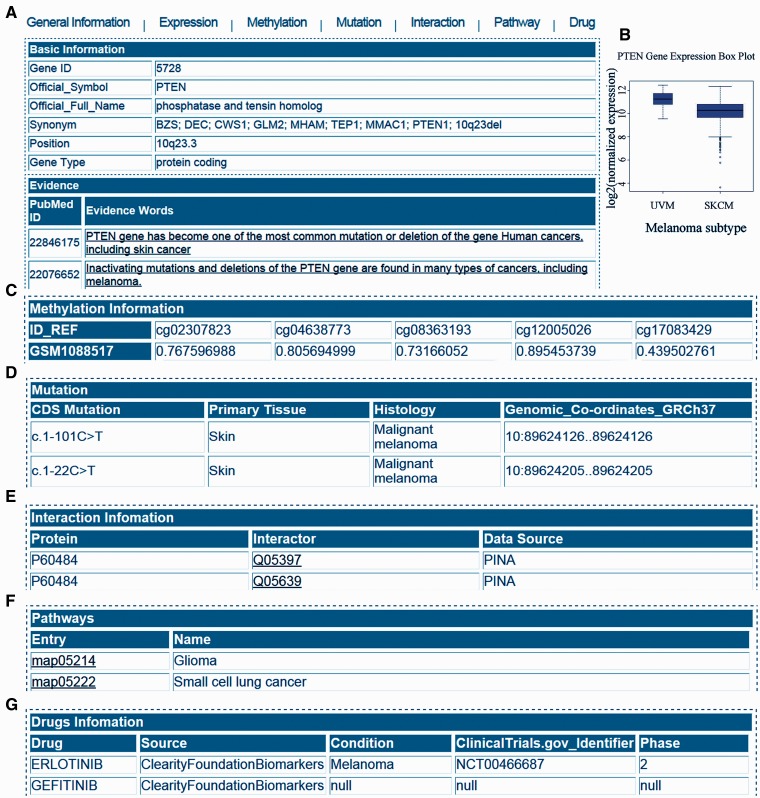
Web interface of MGDB. (**A**) General information in the MGDB. (**B**) Gene expression profile. (**C**) Gene methylation profile. (**D**) Mutation information. (**E**) Interaction information. (**F**) KEGG pathway entry. (**G**) Drugs information.

To better understand the function of these melanoma genes in our database, we collected their extensive functional information. The representative annotations in the MGDB database are summarized in Table 1, which includes a basic description of the gene, gene expression, methylation, somatic mutation information, protein–protein interactions, pathway information and drug information (Figure 1). Details of these databases can be found through the cited references as well as from MGDB.

**Table 1.** Annotation entry statistics for 527 melanoma genes

Data category	Related entries	Annotated melanoma genes	Content/sources
Human Melanoma genes	527	527	Gene names, full name, genomics position, synonym, denition from Entrez gene database
Literature	9788	997	Literature evidence for Human Melanoma genes
Expression of protein-coding genes	420	420	Expression in 470 SKCM^a^ samples and 80 UVM^a^ from TCGA database
Expression of Non-coding genes	89	89	Expression in 452 SKCM^a^ samples and 80 UVM^a^ from TCGA database
Methylation	1926	396	Methylation in melanoma-related samples from GEO (16)
Genomic variation	2418	300	COSMIC database
Protein-Protein Interaction	10 667	352	PINA database
Pathway	1833	185	KEGG database
Drug	1236	133	DGIdb database

^a^SKCM is short for Skin Cutaneous Melanoma, and UVM is short for Uveal Melanoma.

## Biological features of 422 protein-coding genes in MGDB

As the majority of melanoma genes are protein-coding genes (422 human protein-coding genes in Supplementary Table S1), we performed the pathway enrichment analysis to investigate their involved pathways using the KOBAS server (21). Statistically significant pathways were determined by a corrected *P* < 0.05 (Benjamini–Hochberg corrected hypergeometric test). Pathways playing important roles in the pathogenesis of melanoma are at the top enriched pathway list, such as ‘Pathways in cancer’, ‘Melanoma’, ‘Signaling by SCF-KIT’, ‘Oncogene Induced Senescence’, ‘p53 signaling pathway’, and ‘Downstream signal transduction’ (Supplementary Table S3).

To further investigate biological processes, we conducted enrichment tests on 422 human protein-coding genes using the online tool DAVID (Supplementary Table S4) (22). We selected those GO terms or InterPro domains with an adjusted *P* < 0.05 (Benjamini–Hochberg corrected hypergeometric test). Using the online DAVID server, 422 protein-coding genes were markedly overrepresented in regulation of cell proliferation, anatomical structure development and developmental process according to GO Biological Processes terms (Table 2). In addition, we observed that the pigmentation relevant GO Biological Processes are enriched in 422 protein-coding genes, including ‘pigmentation during development’, ‘pigmentation’ and ‘regulation of pigmentation during development’ (Supplementary Table S4). These results highlight that these biological processes may be involved in transition of pigment cell progression to cancerous state. Lastly, assignment of 422 genes to InterPro domains (Supplementary Table S4) revealed that the top four categories represented in the dataset are: ‘Protein kinase, core’, ‘Protein kinase, ATP binding site’, ‘Tyrosine protein kinase’ and ‘Tyrosine protein kinase, active site’, thereby suggesting importance of kinase activity in melanoma cells.

**Table 2.** Top 20 enriched GO biological processes of the 422 protein-coding genes

GO term	*P* value	Benjamini–Hochberg corrected *P* value
Regulation of cell proliferation	3.51672E−50	1.18936E−46
Anatomical structure development	9.37996E−49	1.58615E−45
Developmental process	9.44707E−48	1.065E−44
System development	1.36888E−47	1.15739E−44
Organ development	5.81004E−46	3.92991E−43
Positive regulation of cellular process	7.13936E−46	4.02422E−43
Positive regulation of biological process	7.30291E−45	3.52835E−42
Multicellular organismal development	6.23231E−42	2.63471E−39
Negative regulation of cellular process	1.30539E−41	4.90536E−39
Negative regulation of biological process	1.5792E−41	5.34085E−39
Cell differentiation	9.92816E−36	3.05246E−33
Cellular developmental process	5.6154E−35	1.58261E−32
Regulation of cell death	4.45433E−34	1.15881E−31
Regulation of programmed cell death	1.83543E−33	4.43387E−31
Response to chemical stimulus	2.92002E−33	6.58366E−31
Regulation of apoptosis	4.54869E−33	9.61479E−31
Multicellular organismal process	1.16316E−31	2.314E−29
Anatomical structure morphogenesis	1.91276E−31	3.59387E−29
Regulation of cell differentiation	1.72459E−30	3.06978E−28
Negative regulation of cell proliferation	4.72448E−30	7.98909E−28

## Prioritization of protein-coding genes and its enriched dense network module

To further assess the importance of each protein-coding gene in MGDB, we performed gene prioritization analysis using the ToppGene web server (23). ToppGene requires two types of gene set, i.e. training gene set, and test gene set. The training gene set contains already well-known melanoma genes, while the test gene set are the remaining interesting genes in MGDB. Here, we compiled a training gene list that included 10 well-known melanoma genes (*BRAF*, *NRAS*, *CDK4*, *MITF*, *PTEN*, *CDKN2A*, TP53, *KIT*, *GNAQ* and *GNA11*). ToppGene ranks candidate genes (test genes) based on functional similarity to training gene list through integration of various types of data such as gene expression, regulatory information, and literature mining data. Based on the gene ranking results of ToppGene (Supplementary Table S5), the top ranked genes tend to have multiple evidences from different data sources. Besides 10 well-studied melanoma genes in the training set, *CDKN1B* was top ranked melanoma gene in remaining 412 genes from the test set. Functional analyses on the top 100 melanoma genes show similar functional distribution with the total 422 melanoma genes. These highly overlapping relationships of top ranked genes support the accuracy of our data.

**Figure 2.**
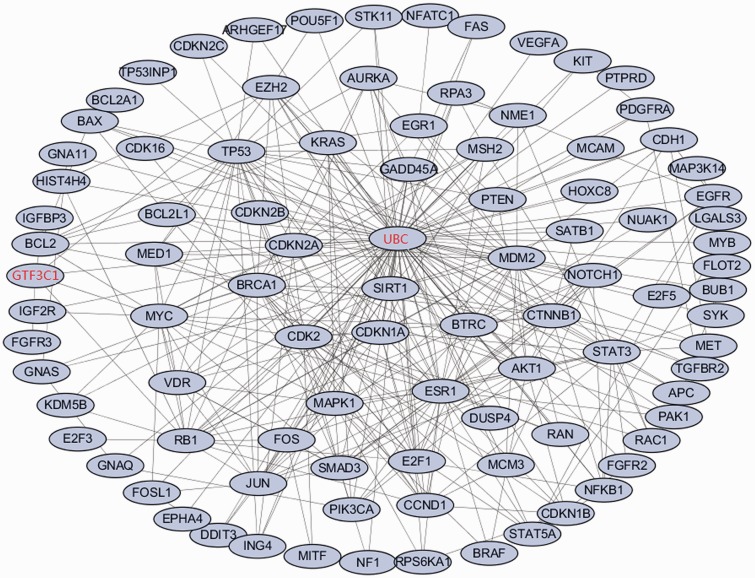
The enriched dense network module for the top ranked melanoma-related genes based on proteinprotein interaction data. The 92 genes in black are genes from the top 100 ranked melanoma-related genes. The remaining two genes in red are the linker genes that bridge the 92 genes.

Employing the software GenRev (24), we further searched for dense modules enriched with the top 100 melanoma associated genes through their protein–protein interactions (PPIs) and identified 1 module comprising of 94 genes (Figure 2). Of the 94 genes, 92 of them are from the top 100 melanoma associated genes. The remaining two are *GTF3C1* and *UBC*. Previous study have found that *GTF3C1* was differentially expressed between melanocytic nevi with and without the V600E BRAF Mutation (25), which may play an important role in melanoma cell. Network analysis highlights *UBC*, a polyubiquitin precursor, as a hub gene in the module, which suggests that the majority of melanoma protein-coding genes are involved in ubiquitination pathway via interacting with *UBC* (26). Frequent alteration of ubiquitination process has been reported in human cancer (27). In conclusion, the majority of the top ranked melanoma-related genes connects each other and forms a dense network, which may further confirm the importance of the gene ranking results.

## Biological features of microRNAs in MGDB

The majority of non-coding genes in MGDB are belonging to microRNAs (miRNAs). We retrieved these miRNAs (Supplementary Table S2) predictive targets from miRTarBase (version 4.5, November 2013) (28). We required that a functional miRNA target interaction was limited in human species, which generated 748 target genes from 84 miRNAs (Supplementary Table S6). Among of the 748 genes, 117 target genes were overlapped with the 422 protein-coding genes in MGDB. Using the KOBAS server (21), we found that these genes are enriched in cancer related pathways, such as melanoma pathway (Supplementary Table S7).

To obtain more reliable miRNA-target genes, we also extracted 202 target genes for functional analysis that were regulated by at least 2 miRNA melanoma genes (Supplementary Table S7). We can observe that 2.97% of the target genes are involved in pigmentation.

## Database construction and web interface

### Database organization

We stored and managed all the data in MySQL (version 5.1.73), which is a popular and open source widely used in biomedical database development. A user-friendly web interface for browsing and searching implemented by JavaScript, the main function were shown in Figure 1, which is freely and systematically retrieve the information.

### Data search

MGDB supports versatile searching, including quick search, advanced search and BLAST search. Firstly, a quick search in the Home page was provided for searching by gene ID or gene names. Secondly, advanced search was incorporated to query gene by filtering multiple options (i.e. gene ID, gene type, official symbol, position or full name) in the Search page. In addition, a search interface to access melanoma genes related literatures provided a window for users to find more comprehensive melanoma genes descriptions from original literature sources. Thirdly, user can utilize an online BLAST interface to input an interesting sequence in FASTA format and query against the melanoma gene nucleotide or protein sequences in our MGDB. Lastly, using the chromosome browser, users can obtain melanoma gene lists that include a summary of the genes and hyperlinks to detailed gene evidence and annotation pages.

### Representative entry in MGDB

The output page displays comprehensive information about the gene entries searched by the user. Figure 1A shows a sample output page generated by querying MGDB using the keyword ‘PTEN’. Annotations of each gene can be obtained by clicking the label ‘General Information’, ‘Expression’, ‘Methylation’, ‘Mutation’, ‘Interaction’, ‘Pathway’ and ‘Drug’ on top of the output page.

In the ‘General information’ page, gene ID, gene official symbol, gene full name, synonym, gene location on chromosome and gene type are displayed. Besides, evidence section includes the literature evidence and the related PubMed ID. Users can click on the hyperlinks of literature evidence to access corresponding PubMed page. DNA, cDNA, and protein sequence can also be found in a tabular view in the ‘General Information’ page. In the ‘Expression’ page, MGDB collects RNA-seq-based level 3 expression data from TCGA. Output in MGDB is a box plot which shows the two melanoma subtypes [Skin Cutaneous Melanoma (SKCM) and Uveal Melanoma (UVM)] expression levels (Log 2 fold change of normalized counts). In the ‘methylation’ page, we use the textual interfaces to demonstrate the methylation patterns in melanoma tissues. In the ‘mutation’ page, full mutation details have been curated from COSMIC (17). Genomic variants in a specific gene were described for its genomic information and the corresponding histology of melanoma. In the ‘interaction’ page, the interaction table for storing gene corresponding UniProt accession number and its interacting partner, which extracted from PINA database (18). In the ‘pathway’ page, genes within its involved pathways were described. Through the entry, we can link to the corresponding pathway in KEGG database (19). In the ‘Drug’ page, we extracted the gene targeted drug information through integrating the DGIdb database (20).

## Conclusions and perspectives

In this study, we applied a comprehensive literature mining method to extract 422 protein-coding genes, 95 microRNAs and 10 lncRNAs in melanoma. MGDB is the first literature-based melanoma database based on literature extraction by integrating multi-dimensional data of genomic, epigenetic, pathway and drug information. It provides a user-friendly web interface, where users can easily access to the database resources through text querying, sequence searching and so on. We believed that MGDB will greatly facilitate cancer researchers’ effort of surveying the literature on genes and their involvement in melanoma. MGDB will be routinely updated to follow research progresses of melanoma biology. We anticipated that MGDB could provide a valuable resource for further studies and understanding of melanoma genetics.

## Supplementary Data

Supplementary data are available at *Database* Online.
